# Common mental disorders in asylum seekers and refugees: umbrella review of prevalence and intervention studies

**DOI:** 10.1186/s13033-017-0156-0

**Published:** 2017-08-25

**Authors:** Giulia Turrini, Marianna Purgato, Francesca Ballette, Michela Nosè, Giovanni Ostuzzi, Corrado Barbui

**Affiliations:** Cochrane Global Mental Health and WHO Collaborating Centre for Research and Training in Mental Health and Service Evaluation, Department of Neuroscience, Biomedicine and Movement Sciences, Section of Psychiatry, University of Verona, Piazzale L.A. Scuro, 10, 37134 Verona, Italy

**Keywords:** Mental health, Asylum seeker, Refugee, Prevalence, Efficacy, Intervention

## Abstract

**Background:**

In recent years there has been a progressive rise in the number of asylum seekers and refugees displaced from their country of origin, with significant social, economic, humanitarian and public health implications. In this population, up-to-date information on the rate and characteristics of mental health conditions, and on interventions that can be implemented once mental disorders have been identified, are needed. This umbrella review aims at systematically reviewing existing evidence on the prevalence of common mental disorders and on the efficacy of psychosocial and pharmacological interventions in adult and children asylum seekers and refugees resettled in low, middle and high income countries.

**Methods:**

We conducted an umbrella review of systematic reviews summarizing data on the prevalence of common mental disorders and on the efficacy of psychosocial and pharmacological interventions in asylum seekers and/or refugees. Methodological quality of the included studies was assessed with the AMSTAR checklist.

**Results:**

Thirteen reviews reported data on the prevalence of common mental disorders while fourteen reviews reported data on the efficacy of psychological or pharmacological interventions. Although there was substantial variability in prevalence rates, we found that depression and anxiety were at least as frequent as post-traumatic stress disorder, accounting for up to 40% of asylum seekers and refugees. In terms of psychosocial interventions, cognitive behavioral interventions, in particular narrative exposure therapy, were the most studied interventions with positive outcomes against inactive but not active comparators.

**Conclusions:**

Current epidemiological data needs to be expanded with more rigorous studies focusing not only on post-traumatic stress disorder but also on depression, anxiety and other mental health conditions. In addition, new studies are urgently needed to assess the efficacy of psychosocial interventions when compared not only with no treatment but also each other. Despite current limitations, existing epidemiological and experimental data should be used to develop specific evidence-based guidelines, possibly by international independent organizations, such as the World Health Organization or the United Nations High Commission for Refugees. Guidelines should be applicable to different organizations of mental health care, including low and middle income countries as well as high income countries.

**Electronic supplementary material:**

The online version of this article (doi:10.1186/s13033-017-0156-0) contains supplementary material, which is available to authorized users.

## Background

Increasing number of people are leaving their country of origin because of human rights violations, persecutions and conflicts. Europe is the largest host continent of people who have been forced to migrate: during 2016, 347,000 refugees and migrants have arrived in Europe, in addition to the over one million refugees and migrants that undertook the perilous journey across the Mediterranean Sea in 2015 [1]. The stressful experiences that many asylum seekers and refugees are exposed to during forced migration, and during the resettlement process, make them vulnerable to mental health conditions, including post-traumatic stress disorder (PTSD), major depression, and anxiety. As a consequence, the prevalence of psychological distress and mental disorders in asylum seekers and refugees appears to be generally high, though estimates vary widely. A number of factors, such as migration exposure to violence, torture, and other potentially traumatic events [2], as well as migration and post-migration factors, like life threatening conditions while traveling to resettlement countries, uncertainty about asylum application and reduced social integration, can affect the mental health of this already vulnerable population, and may influence the rate of mental health conditions [3, 4].

Addressing mental health in this population is demanding. Despite a growing body of evidence on the effectiveness of psychosocial and pharmacological intervention, this field is characterized by different and heterogeneous approaches and remains relatively confused. Therefore, the provision of evidence-based mental health interventions constitutes a particular challenge for health care systems.

Against this background, we examined the evidence on the rates and characteristics of common mental disorders and on the efficacy of both psychosocial and pharmacological interventions, in adult and children asylum seekers and refugees, resettled in low-, middle, and high-income countries. We conducted an umbrella review, that is a systematic collection and critical appraisal of multiple systematic reviews performed on this topic.

## Methods

### Data source and search strategy

The protocol for this review was registered in the International Prospective Register of Systematic Reviews (PROSPERO), Registration Number: CRD42017056338. A systematic search of the literature was conducted from inception to April 2017 in order to identify (a) systematic reviews summarizing the prevalence of common mental disorders in adult and children asylum seekers and/or refugees resettled in low, middle, and high income countries, and (b) systematic reviews summarizing the efficacy of psychosocial and pharmacological interventions in the same population with any mental health disorders. The search was conducted on the following databases: Cochrane database of systematic reviews, MEDLINE, Web of Science, PubMed, PsycINFO, Embase, CINAHL and WHO Global Health Library. The McMaster University algorithm to locate systematic reviews was used [5] and complemented with the terms asylum seeker*, refugee*, migrant*, immigrant* AND mental health, mental disorder, mental illness, psych*, mental*.

The inclusion criteria were: systematic reviews with a quantitative or qualitative summary of the prevalence of common mental disorders and systematic reviews on the efficacy of psychosocial and/or pharmacological interventions; systematic search for primary studies; participants were adult and/or children asylum seekers and/or refugees; the setting was a low, middle or high-income country. Only systematic reviews published in English were considered. Reference lists of all eligible review articles were hand-searched to identify additional eligible systematic reviews. The selection process was recorded in agreement with the Preferred Reporting Items for Systematic Reviews and Meta-Analyses (PRISMA) [6].

Two review authors (GT and MP) independently screened titles and abstracts for inclusion. Articles rated as possible candidates by either of the two reviewers were added to a preliminary list and their full texts were retrieved. Working independently and in duplicate, the two review authors inspected the full texts for inclusion. Discrepancies between reviewers were resolved by consensus or through discussion with the research team.

### Quality assessment

Review quality was rated independently by two reviewers using AMSTAR (A Measurement Tool to Assess Systematic Reviews), an 11-point assessment tool of the methodological quality of systematic reviews criteria. AMSTAR has good inter-rater agreement, test-retest reliability and content validity [7]. It assesses reviews on the following categories: (1) A priori design provided; (2) duplicate study selection and data extraction; (3) comprehensive literature search; (4) status of publication used as an inclusion criterion; (5) list of studies provided (included and excluded); (6) characteristics of studies provided; (7) scientific quality of studies assessed; (8) scientific quality used appropriately in formulating conclusions; (9) appropriate methods used to combine study findings; (10) likelihood of publication bias assessed; (11) conflict of interest included. According to AMSTAR, a total score of 0–4 indicates low quality; a score of 5–8 moderate quality; and a score 9–11 high quality.

### Data extraction and presentation

For each included systematic review, we extracted information on the year and country of publication, review aims, characteristics of the included studies, settings and populations, type of experimental and control interventions, and summary results (overall estimates for meta-analyses and qualitative results for systematic reviews without a meta-analysis). Data extraction was performed independently by two investigators (GT and FB), and in case of discrepancies, a consensus was reached by a third investigator (CB). Systematic review findings were presented in a tabular form and descriptively, because of the expected heterogeneity between studies, and because most results of included reviews were reported in a narrative form. For systematic reviews presenting quantitative findings on the prevalence of common mental disorders, overall estimates with 95% confidence intervals were extracted and graphically presented in forest plots. For systematic reviews of intervention studies, specific symbols were used to visually represent study findings. For systematic reviews of both prevalence and intervention studies, results were presented by type of mental health outcome, a priori focusing on the following: PTSD, anxiety, depression.

## Results

**Fig. 1:**
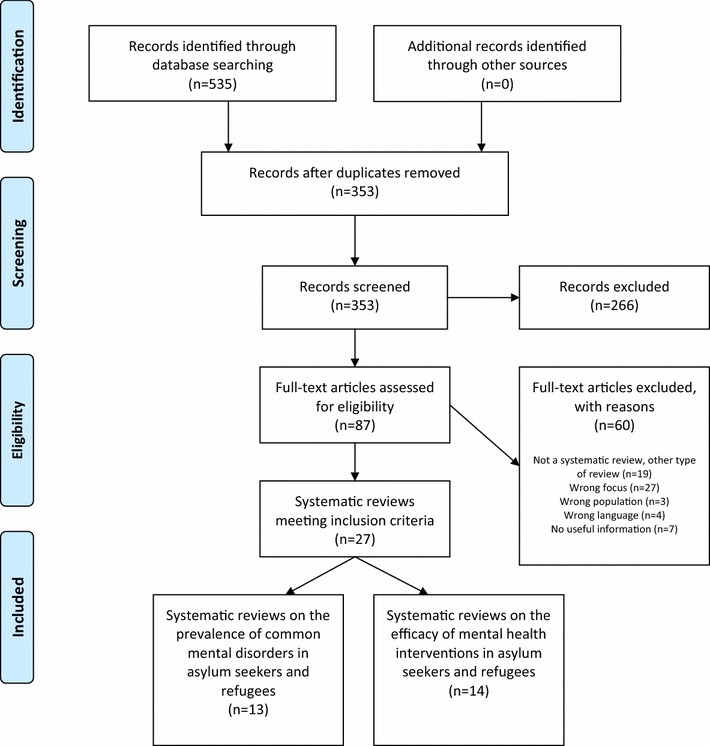
PRISMA flow-chart diagram

The electronic search yielded a total number of 535 records. After removing duplicates, 353 records undergo the title and abstract screening. After this phase, 87 full text papers were considered for inclusion. Twenty-seven systematic reviews met the inclusion criteria and were included in this umbrella review [2, 8–33] (see Fig. 1). Thirteen reviews reported data on the prevalence of PTSD or trauma-related symptoms, anxiety and depressive symptoms, while fourteen reviews reported data on the efficacy of psychological or pharmacological interventions. Only three reviews [2, 8, 9] employed meta-analytical techniques to pool prevalence rates or intervention effects.

### Quality assessment of included studies

Of the 27 reviews, three were of high quality according to the AMSTAR scoring system, 11 were of moderate quality and 13 received a low quality rating (see Table 1). Of the 13 low quality reviews, four reported data on the prevalence of mental disorders and nine covered the efficacy of interventions. AMSTAR detected that in most reviews a study protocol was not available, study selection and data extraction were not performed in duplicate, inclusion of grey literature and unpublished studies was unclear, a list of included and excluded studies was not provided, risk of bias of primary studies was not assessed, publication bias and conflicts of interest were not taken into account as potential confounders. In addition, only a minority of reviews reported the reasons why pooling of primary study findings was deemed as appropriate or inappropriate, based clinical or statistical heterogeneity. Details of the AMSTAR items for each included review are reported in Table 1.

**Table 1:** Quality rating of included systematic reviews based on the AMSTAR tool

Study	1. Priori design provided	2. Duplicate study selection and data extraction	3. Comprehensive literature search	4. Status of publication an inclusion criteria	5. List of studies provided (included and excluded)	6. Characteristics of studies provided	7. Scientific quality of studies assessed	8. Scientific quality used appropriately in formulating conclusions	9. Appropriate methods used to combine study findings	10. Likelihood of publication bias assessed	11. Conflict of interest included	Overall quality rating
Prevalence												
Alemi et al. [10]	✘	✘	✘	✘	✔	✔	✔	✘	✔^b^	✘	✔	5/11 (moderate)
Bogic et al. [17]	✔^a^	✔	✔	✔	✘	✔	✔	✔	✔^b^	✔	✔	10/11 (high)
Bronstein and Montgomery [13]	✔	✘	✔	✔	✘	✔	✔	✔	✔^b^	✘	✘	7/11 (moderate)
Fazel et al. [8]	✘	✘	✔	✔	✘	✔	✘	✘	✔	✘	✔	5/11 (moderate)
Keyes [18]	✘	✘	✔	✘	✘	✔	✘	✘	✘	✘	✘	2/11 (low)
Lindert et al. [9]	✔	✘	✘	✘	✘	✔	✘	✘	✔	✘	✘	3/11 (low)
Mills et al. [14]	✘	✔	✔	✔	✘	✔	✘	✘	✔^b^	✘	✔	6/11 (moderate)
Mills et al. [15]	✘	✔	✔	✔	✘	✔	✘	✘	✔^b^	✘	✘	5/11 (moderate)
Quosh et al. [16]	✘	✘	✔	✔	✘	✔	✘	✘	✔^b^	✘	✘	4/11 (low)
Robjant et al. [11]	✘	✘	✔	✘	✘	✔	✘	✘	✘	✘	✔	3/11 (low)
Slewa-Younan et al. [12]	✘	✔	✔	✘	✘	✔	✔	✔	✘	✘	✘	5/11 (moderate)
Steel et al. [2]	✘	✘	✔	✘	✔	✔	✘	✘	✔	✔	✔	6/11 (moderate)
Storm and Enberg [19]	✘	✔	✔	✘	✘	✔	✘	✘	✔^b^	✘	✔	5/11 (moderate)
Intervention												
Crumlish and O’Rourke [21]	✘	✘	✔	✔	✔	✔	✔	✔	✔^b^	✘	✘	7/11 (moderate)
Mc Farlane and Kaplan [22]	✘	✘	✔	✘	✘	✔	✔	✘	✔^b^	✘	✘	4/11 (low)
Nickerson et al. [23]	✘	✘	✔	✘	✔	✔	✘	✘	✘	✘	✘	3/11 (low)
Nosè et al. [20]	✔	✘	✔	✔	✔	✔	✔	✔	✔	✔	✔	10/11 (high)
Palic and Elklit [24]	✘	✘	✔	✘	✔	✔	✔	✘	✔^b^	✘	✔	8/11 (moderate)
Patel et al. [25]	✔	✔	✔	✔	✔	✔	✔	✔	✔	✔	✔	11/11 (high)
Peltonen and Punamäki [26]	✘	✘	✘	✘	✘	✔	✔	✔	✔	✘	✘	4/11 (low)
Robjant and Fazel [27]	✘	✘	✔	✔	✘	✔	✘	✘	✘	✘	✘	3/11 (low)
Slobodin and de Jong [28]	✘	✘	✔	✘	✘	✔	✘	✘	✘	✘	✔	3/11 (low)
Sonne et al. [29]	✘	✘	✔	✘	✘	✔	✘	✘	✔^b^	✘	✔	4/11 (low)
Sullivan and Simonson [30]	✘	✘	✔	✘	✘	✔	✘	✘	✔^b^	✘	✘	3/11 (low)
Tyrer and Fazel [31]	✘	✘	✔	✔	✘	✔	✔	✔	✔^b^	✘	✔	7/11 (moderate)
van Wyk and Schweitzer [32]	✘	✘	✔	✘	✘	✔	✘	✘	✘	✘	✘	2/11 (low)
Williams and Thompson [33]	✘	✘	✔	✘	✘	✔	✔	✘	✘	✘	✘	3/11 (low)

^a^A protocol is mentioned in the text, but it is not available/accessible

^b^The authors reported that between-study heterogeneity did not allow meta-analysis of results

### Prevalence studies in populations resettled in high-income countries

Five systematic reviews summarized the prevalence of PTSD, depression and anxiety in asylum seekers and/or refugees resettled in high income countries [8, 10–13]. The review carried out by Alemi and colleagues [10] included 17 studies (1250 participants) that described Afghan populations of any age. It reported higher rates of depression (range 55–57%) than PTSD and anxiety (see Table 2). Similar results were obtained by Robjant and colleagues [11], who included 10 studies (877 participants) that described asylum seekers and refugees resettled in Australia, UK and USA. Depression ranged between 59 and 100%, anxiety was found in 77%, and PTSD in 27–50% of the whole population. Slewa-Younan and colleagues [12], who included 8 studies (2148 participants) of adult Iraqi asylum seekers or refugees resettled in western countries, reported an average rate of depression of 43%, and a rate of PTSD of 25%. In children and adolescents, by contrast, Bronstein and Montgomery [13] included 22 studies (3003 participants) that provided an average frequency of 18% for depression and 36% for PTSD. The review of Fazel and colleagues (25 studies, 7003 participants) [8], focused on refugees of all age groups resettled in western countries, suggested slightly higher frequency for PTSD (9%) than depression (5%) and anxiety (4%) in adult population. Additionally, five surveys of 260 children yielded a prevalence of 11% for PTSD and no relevant studies of depression or anxiety were identified.

**Table 2:** Characteristics and main findings of systematic reviews summarizing the prevalence of PTSD, depression and anxiety

Systematic reviews	Target population	No studies (patients)	Mental disorders	Prevalence range	Average
Alemi et al. [10]	Afghan asylum seekers or refugees of all age groups, resettled in western countries, with a length of residence between 3 days and 21 years	17 (1250)	PTSD	25.4–50%	Not reported
			Depression	54.7–57%	
			Anxiety	12–39.3%	
Bogic et al. [17]	Adult war-refugees 5 years or longer after displacement (including a proportion residing outside western countries)	29 (16,010)	PTSD	4.4–86%	Not reported
			Depression	2.3–80%	
			Anxiety	20.3–88%	
Bronstein and Montgomery [13]	Refugee children and adolescents (<25 years) resettled in western countries	22 (3003)	PTSD	19–54%	36.0%
			Depression	3–30%	18.0%
Fazel et al. [8]	Refugees of all age groups resettled in western countries	25 (7003)	PTSD^a^	3–18%	9.0% (99% CI 8.0–10.0%)
			Depression^a^	2–10%	5% (99% CI 4.0–6.0%)
			Anxiety^a^	Not reported	4% (99% CI 3.0–6.0%)
Keyes [18]	Adult and children refugees resettled in low, middle and high income countries	12 (2065)	PTSD	12–86%	Not reported
			Depression	16–80%	
			Anxiety	6%	
Lindert et al. [9]	Adult and adolescent refugees resettled in low, middle and high income countries	35 (24,051)	PTSD	4–68%	36.0% (95% CI 23.0–49.0%)
			Depression	3–81%	44.0% (95% CI 27.0–62.0%)
			Anxiety	5–90%	40.0% (95% CI 17.0–64.0%)
Mills et al. [14]	Adult and children Tibetan refugees in lower middle income country	5 (926)	PTSD	11–23%	Not reported
			Depression	11.5–57%	
			Anxiety	25–77%	
Mills et al. [15]	Bhutanese torture refugees resettled in refugee camps in Nepal	6 (4712)	PTSD	14–43%	Not reported
			Depression	2–25%	
			Anxiety	4–43%	
Quosh et al. [16]	Adult and children Iraqi and Syrian refugees resettled in lower and upper middle income countries	44 (about 65,000)	PTSD	0.2–76.5%	Not reported
			Depression	16.67–89.5%	
			Anxiety	15.6–81.6%	
Robjant et al. [11]	Adult, adolescent or children detained asylum seekers or refugees resettled in Australia, UK, USA	10 (877)	PTSD	27–50%	Not reported
			Depression	59–100%	
			Anxiety	77%	
Slewa-Younan et al. [12]	Adult Iraqi asylum seekers or refugees resettled in western countries	8 (2148)	PTSD	8–37.2%	25.0%
			Depression	28.3–75%	43.0%
Steel et al. [2]	Adult asylum seekers or refugees and other conflict-affected populations resettled in low, middle and high income countries	161 (81,866)	PTSD	0–99%	30.6% (95% CI 26.3–35.2%)
			Depression	3–85.5%	30.8% (95% CI 26.3–35.6%)
Storm and Engberg [19]	Adult detained asylum seekers, torture survivors and refugees resettled in low, middle and high income countries	15 (1716)	PTSD	50–86%	Not reported
			Depression	76.4–100%	
			Anxiety	72–77%	

*CI* confidence interval

^a^Prevalence rates in adult refugees

### Prevalence studies in populations resettled in low- and middle-income countries

Three reviews summarized the prevalence of PTSD, depression and anxiety in asylum seekers and/or refugees of any age resettled in low- and middle-income countries [14–16]. Mills and colleagues included five studies (926 participants) on Tibetan refugees [14], Mills and colleagues included six studies (4712 participants) on Bhutanese refugees [15], and Quosh and colleagues included 44 studies (about 65,000 participants) on Iraqi and Syrian refugees [16]. These reviews found high levels of heterogeneity across the study results, yielding wide and imprecise prevalence ranges that indicated similar rates of depression, PTSD and anxiety (see Table 2).

### Prevalence studies in populations resettled in low-, middle- and high-income countries

Five systematic reviews summarized the prevalence of PTSD, depression and anxiety in asylum seekers and/or refugees resettled in low-, middle-, and high-income countries [2, 9, 17–19]. Bogic and colleagues [17] identified 29 studies (16,010 participants) on adult war-refugees 5 years or longer after displacement. It found prevalence estimates ranging from 20 to 80% for all clinical outcomes, and similar results were found by Keyes et al. [18] in a population of 2065 adult and children refugees. A review by Storm and Engberg [19] included a total of 15 studies (1716 participants) conducted in adult detained asylum seekers, torture survivors and refugees. This review found prevalence estimates above 50% for depression, PTSD and anxiety. Two additional systematic reviews carried out formal meta-analyses of prevalence studies. Lindert and colleagues [9] included 35 studies (24,051 participants) on adolescent and adult refugees, and found a prevalence rate of 44% for depression, 40% for anxiety and 36% for PTSD. Steel and colleagues [2] included 161 studies (81,866 participants) on adult asylum seekers or refugees and other conflict-affected populations, and found a prevalence rate of 31% for both depression and PTSD.

### Overall summary of prevalence studies

**Fig. 2:**
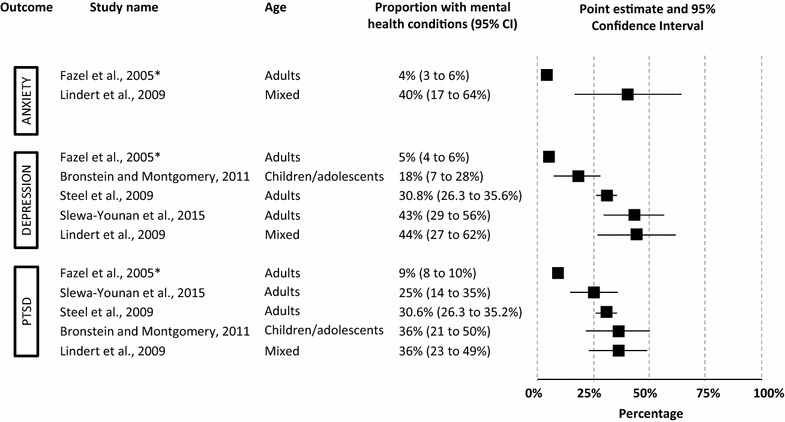
Prevalence rates (%, with 95% CI) as reported by systematic reviews calculating overall summary measures. *A 99% confidence interval was used by Fazel et al. [8]

Five systematic reviews calculated overall summary measures for PTSD, depression and anxiety outcomes [2, 8, 9, 12, 13] (see Fig. 2; Table 2). For each of these outcomes wide differences in point estimates were found (4 to 40% for anxiety, 5 to 44% for depression, 9 to 36% for PTSD).

### Efficacy of interventions on PTSD and trauma-related outcomes

Data on PTSD outcomes or trauma related symptoms were reported in 14 reviews [20–33] (see Table 3). A total of 12 studies (640 participants) investigated the efficacy of narrative exposure therapy (NET). NET was effective in eight randomized clinical trials (RCTs) against inactive interventions, and in two out of three randomized trials against an active control group. In addition to that, one uncontrolled before-and-after study provided positive results. Different forms of cognitive behavioral therapy (CBT) were investigated in 12 studies (455 participants). Four out of six RCTs and two controlled clinical trials (CCTs) supported the efficacy of CBT against inactive interventions, while one RCT against active control failed to show a beneficial effect of this intervention. Additionally, two of three before-and-after studies provided positive results. For other ○interventions, very few and scant data were available (see Table 3).

**Table 3:** Qualitative summary of the efficacy of interventions by type of mental health outcome

Outcomes	Interventions	Comparison	Study design			No of patients	Systematic reviews
			RCTs	CCTs	Pre-post		
PTSD/trauma-related symptoms	NET	Inactive (N = 8)	●●●●●●●●			640	Crumlish and O’Rourke [21]; McFarlane and Kaplan [22]; Nickerson et al. [23]; Nosè et al. [20]; Palic and Elklit [24]; Patel et al. [25]; Robjant and Fazel [27]
		Supportive counseling, stress inoculation training, trauma counselling (N = 3)	●●○				
		Without control group (N = 1)			▲		
	KIDNET	Inactive (N = 1)	●			26	Crumlish and O’Rourke [21]; Robjant and Fazel [27]
	EMDR	Stabilisation (no exposure) (N = 1)	○			20	Patel et al. [25]
	CBT/culturally sensitivity CBT/CPT	Inactive (N = 8)	●●●●○○	■■		455	Crumlish and O’Rourke [21]; McFarlane and Kaplan [22]; Nickerson et al. [23]; Nosè et al. [20]; Palic and Elklit [24]; Peltonen and Punamaki [26]; Sonne et al. [29]; Sullivan and Simonson [30]; Tyrer and Fazel [31]; Williams and Thompson [33]
		Exposure therapy (N = 1)	○				
		Without control group (N = 3)			▲▲△		
	Testimony therapy	Without control group (N = 1)			▲	20	McFarlane and Kaplan [22]; Nickerson et al. [23]; Palic and Elklit [24]
	Trauma-focused therapy	Inactive (N = 2)		■■		197	McFarlane and Kaplan [22]; Nickerson et al. [23]; Nosè et al. [20]; Palic and Elklit [24]; Sullivan and Simonson [30]; Tyrer and Fazel [31]
		Child-centered play therapy (N = 1)	○				
		Without control group (N = 1)			▲		
	Multimodal interventions/multidisciplinary treatments	Without control group (N = 7)			▲▲▲△△△△	248	McFarlane and Kaplan [22]; Nickerson et al. [23]; Palic and Elklit [24]; Peltonen and Punamaki [26]; Slobodin and de Jong [28]; Sullivan and Simonson [30]; Tyrer and Fazel [31]; Van Wyk and Schweitzer [32]
	Antidepressants^a^	Without control group (N = 1)			▲	32	Crumlish and O’Rourke [21]; Sonne et al. [29]
	Antidepressants^b^ + supportive therapy	Without control group (N = 2)			▲▲	68	Sonne et al. [29]
	Psychotropic medication + psychodinamic therapy	Inactive (N = 1)				50	Palic and Elklit [24]; Sonne et al. [29]
Anxiety symptoms	NET	Inactive (N = 1)	○			43	Crumlish and O’Rourke [21]; Nickerson et al. [23]; Palic and Elklit [24]; Robjant and Fazel [27]
		Supportive counseling (N = 1)	○				
	EMDR	Stabilisation (no exposure) (N = 1)	○			20	Patel et al. [25]
	CBT/culturally sensitivity CBT/CPT	Inactive (N = 4)	●●●			189	Crumlish and O’Rourke [21]; McFarlane and Kaplan [22]; Nickerson et al. [23]; Palic and Elklit [24]; Peltonen and Punamaki [26]; Sonne et al. [29]; Sullivan and Simonson [30]; Tyrer and Fazel [31]
		Exposure therapy (N = 1)	○	■			
		Without control group (N = 2)			▲△		
	Multimodal interventions/multidisciplinary treatments	Without control group (N = 6)			▲▲△△△△	218	McFarlane and Kaplan [22]; Nickerson et al. [23]; Palic and Elklit [24]; Peltonen and Punamaki [26]; Slobodin and de Jong [28]; Van Wyk and Schweitzer [32]; Tyrer and Fazel [31]
Depressive symptoms	NET	Inactive (N = 5)	●●○○○	□		250	Crumlish and O’Rourke [21]; McFarlane and Kaplan [22]; Nickerson et al. [23]; Nosè et al. [20]; Palic and Elklit [24]; Patel et al. [25]; Robjant and Fazel [27]
		Supportive counseling, stress inoculation training (N = 2)	○○				
		Without control group (N = 1)			▲		
	EMDR	Stabilisation (no exposure) (N = 1)	○			20	Patel et al. [25]
	CBT/culturally Sensitivity CBT/CPT	Inactive (N = 5)	●●○○			389	Crumlish and O’Rourke [21]; McFarlane and Kaplan [22]; Nickerson et al. [23]; Nosè et al. [20]; Palic and Elklit [24]; Peltonen and Punamaki [26]; Sonne et al. [29]; Sullivan and Simonson [30]; Tyrer and Fazel [31]
		Exposure therapy (N = 1)	○				
		Without control group (N = 3)			▲▲△		
	Testimony therapy	Without control group (N = 1)			▲	20	McFarlane and Kaplan [22]; Nickerson et al. [23]; Palik and Elklit [24]
	Multimodal interventions/multidisciplinary treatments	Without control group (N = 7)			▲▲▲△△△△	248	McFarlane and Kaplan [22]; Nickerson et al. [23]; Palic and Elklit [24]; Peltonen and Punamaki [26]; Slobodin and de Jong [28]; Sullivan and Simonson [30]; Van Wyk and Schweitzer [32]; Tyrer and Fazel [31]
	Antidepressants^c^	Without control group (N = 1)				32	Crumlish and O’Rourke [21]; Sonne et al. [29]

*NET* narrative exposure therapy, *KIDNET* narrative exposure therapy for children, *EMDR* eye movement desensitisation and reprocessing, *CBT* cognitive behavioral therapy, *CPT* cognitive processing therapy

■ = controlled clinical trial (CCT) showing a statistically significant positive effect,  □ = controlled clinical trial (CCT) failing to show a statistically significant positive effect,  ● = randomised clinical trial (RCT) showing a statistically significant positive effect,  ○ = randomised clinical trial (RCT) failing to show a statistically significant positive effect,  ▲ = study without control group showing a statistically significant positive effect,  △ = study without control group failing to show a statistically significant positive effect

^a^Paroxetine, sertraline and venlafaxine

^b^TCA or MAOI, or a combination of these medications

^c^Only venlafaxine did not show improvement in Beck depression inventory

### Efficacy of interventions on anxiety symptoms

Data on anxiety outcomes were reported in 12 reviews [21–32] (see Table 3). The efficacy of NET was investigated in two RCTs (43 participants) that provided negative results. Different forms of CBT were investigated in 7 studies (189 participants). Three RCTs and one CCT supported the efficacy of CBT against inactive interventions, while one RCT against active control failed to show a beneficial effect of this intervention. Additionally, one of two before-and-after studies provided positive results. For other interventions, very few and scant data were available (see Table 3).

### Efficacy of interventions on depressive symptoms

Data on depressive outcomes were reported in 13 reviews [20–32] (see Table 3). A total of 8 studies (250 participants) investigated the efficacy of NET. NET was effective in two out of five RCTs against inactive interventions, while two RCTs against an active control group failed to show an effect. Additionally, one uncontrolled before-and-after study provided positive results. Different forms of CBT were investigated in 9 studies (389 participants). Two out of four RCTs showed the efficacy of CBT against inactive interventions, while one CCT against inactive control failed to show a beneficial effect of this intervention. Against active control, one RCT did not show any beneficial effect. Additionally, two of three before-and-after studies provided positive results. For other interventions, very few and scant data were available (see Table 3).

### Overall summary of intervention studies

NET and CBT were the most studied interventions. PTSD measures were the most studied outcomes. While the majority of studies against inactive interventions provided positive results, only two of 10 RCTs against active interventions showed a beneficial effect of the experimental interventions. Uncontrolled before-and-after studies generated unclear and contrasting findings. A qualitative summary of the study results is reported in Additional file 1.

## Discussion

In this umbrella review we summarized the prevalence rates of common mental disorders, specifically PTSD, depression and anxiety, and the efficacy of psychosocial and pharmacological interventions in adult and children asylum seekers and refugees. We found substantial heterogeneity in prevalence rates, ranging from low to very high proportions. This variability may be explained by a number of factors, both methodological and clinical. Methodologically, different designs with different sampling strategy were adopted by the primary studies. A major aspect was that the diagnostic criteria were not consistent across studies: different tools were used, including structured interviews and self-report questionnaires, whose validity was sometimes rather questionable. Furthermore, most of the instruments used were based on Western notions of mental health and illness, and this may have led to a misunderstanding of the symptoms experienced by non-western populations [34, 35]. Clinically, the reviews summarized here compared very different cultural groups from different countries and resettled in different host countries, with different reason for migration, different exposure to post migration stressors and very different lengths of stay in the host country. These factors and/or their complex interaction may had a crucial role in shaping individuals’ behavior and mental health [36]. For these reason, the high variability of findings among reviews, may reflect true clinical, social, personal and context differences.

Because of such heterogeneity, most systematic reviews did not employ meta-analytical techniques. However, when meta-analysis was possible, overall prevalence rates clearly indicated that rates of depression and anxiety were as high as rates of PTSD, affecting on average one out of three asylum seekers and refugees. This finding is of paramount relevance not only to generally emphasize the epidemiological relevance of common mental disorders in this group, but also to specifically underline that depression and anxiety disorders should receive careful clinical and policy consideration. This aspect may be important in a field that is currently dominated by programs and research activities that are almost entirely focused on PTSD.

Given the epidemiological relevance of these conditions, it was important to highlight that a diverse array of mental health interventions has been employed to enhance mental health and reduce the symptoms of PTSD, depression and anxiety in asylum seekers and refugees. However, the primary studies included in the reviews summarized here had a number of methodological limitations. Lack of a control condition was a major issue in several before-and-after intervention studies, limiting the conclusions about the efficacy of interventions. This was especially true for multimodal interventions, which encompass a variety of components that typically include general resettlement assistance, medical care and psycho-therapeutic interventions, and pharmacological treatments. Before-and-after studies were also difficult to interpret, as it was not possible to clearly ascertain whether improvements were related to the provision of specific treatments or, rather, to a general phenomenon of regression to the mean. With regards to RCTs, we noted that the majority of these studies were focused on PTSD outcomes in participants exposed to NET and other forms of CBT. Positive results in favor of these psychological interventions were found only against inactive interventions, while against active control groups no clear differences emerged.

In terms of overall findings, qualitatively, it was possible to describe that NET and CBT were supported by evidence of efficacy in this group, especially on PTSD outcomes. Quantitatively, the only review that employed meta-analytical techniques [20] showed that, overall, psychosocial interventions were effective in decreasing PTSD symptoms relative to control groups, with a standardised mean difference of −1.03, and a 95% confidence interval ranging from −1.55 to −0.51. The corresponding number needed to treat was between 4 and 5 patients, suggesting that one in 4–5 patients exposed to psychosocial interventions would show a clinically significant beneficial effect as compared to the control group. Interestingly, despite the high frequency of depression and anxiety, only a minority of the included studies collected data on the efficacy of interventions on these outcome measures.

We acknowledge that this overview has limitations. In general, the considerations reported above on the heterogeneity of the primary studies included in the reviews represent a limitation of the present overview. In particular we were not able to extract from the included reviews basic information on important aspects, such as from which countries refugees mainly come from and in which host countries they resettled. A second limitation is that we were able to present quantitative figures for a selection of prevalence studies only, with a risk of selection bias, while for intervention studies only a qualitative description of findings was possible. Although we acknowledge this as a limitation, we reasoned that it was important to give visibility to the whole range of intervention studies, including those with weak study designs, as they may be important to inform a new generation of high-quality research in the field. A further limitation is that the current overview systematically examined the evidence focusing only on PTSD, depression and anxiety, the best studied mental health outcomes in this population, but did not cover other mental health conditions due to the lack of sufficient data and reviews focusing on other outcomes.

This overview has important implications. First, it clearly shows that current evidence base needs to be expanded with more rigorous studies focusing not only on PTSD, but also on a wider range of outcomes, including depression, anxiety and comorbid medical conditions that are frequent in this population. For example, Bartoli and colleagues [37] found that people suffering from PTSD have significantly higher risk of obesity and metabolic disorders than the general population. We therefore need research activities assessing possible relationships between physical illness and mental conditions. Moreover, new studies are urgently needed to assess the efficacy of psychosocial interventions when compared to each other, evaluating which factors contribute to positive treatment outcomes and how interventions might be adapted based on refugees’ needs. In the general population of adults and children with PTSD, existing guidelines suggest an individual or group CBT with a trauma focus, or eye movement desensitization and reprocessing (EMDR), with the addition of stress management interventions for adult. Additionally, selective serotonin re-uptake inhibitors and tricyclic antidepressants are suggested for adults if psychosocial interventions have failed or if there is co-morbid moderate to severe depression [38]. We note that these guidelines are focused on PTSD only, and do not specifically cover the mental health needs of asylum seekers and refugees. Similarly, the National Institute for Health and Care Excellence (NICE) guidance for the management of PTSD [39] and the World Health Organization Mental Health Gap Action Programme Humanitarian Intervention Guide (WHO mhGAP-HIG) [40], are only indirectly relevant to this population, as they include recommendations that do not respond directly to the specific needs of this vulnerable and complex population. In asylum seekers and refugees, the report by Priebe and colleagues [41] suggested some policy options that European countries might employ in order to support policy-makers in strengthening or introducing specific policies regarding mental health care for migrants, but no evidence-based guidelines were formulated. In Italy specific guidelines were recently issued at a national level for the treatment of tortured refugees’ mental health [42].

Related to this, in situation of humanitarian crisis, the Interagency Standing Committee issued guidance on Mental Health and Psychosocial Support in Emergency Settings to enable humanitarian actors to plan, establish and coordinate a set of minimum multi-sectoral and multi-layered responses to protect and improve people’s mental health and psychosocial well-being [43]. This approach appears to be promising and highly pragmatic, and might be used to develop similar actions applicable to asylum seekers and refugees. General good practice principles were also developed for asylum-seekers, refugees and migrants in Europe by United Nations High Commissioner for Refugees (UNHCR), International Organization for Migration (IOM) and Mental Health and Psychosocial Support Network (MHPSS) [44].

## Conclusions

Given the pressing mental health needs of asylum seekers and refugees, and in view of the existing data on the epidemiological relevance of common mental disorders and on the effectiveness of some psychosocial interventions, we argue that specific evidence-based guidelines should be developed, possibly by international independent organizations, such as the WHO or the United Nations High Commission for Refugees. Guidelines should be applicable to different organizations of mental health care, including low and middle income countries as well as high income countries, and should be implemented to ensure that all people have equitable access to high-quality mental health care.

## Additional file

**Additional file 1.** Overall summary of the efficacy of interventions.

## Abbreviations

PTSD: posttraumatic stress disorder
PRISMA: preferred reporting items for systematic reviews and meta-analyses
AMSTAR: a measurement tool to assess systematic reviews
NET: narrative exposure therapy
CBT: cognitive behavioral therapy
KIDNET: narrative exposure therapy for children
EMDR: eye movement desensitization and reprocessing
CBT: cognitive behavioral therapy
CPT: cognitive processing therapy
RTCs: randomized controlled trials
CCTs: controlled clinical trials
NICE: The National Institute for Health and Care Excellence
WHO: World Health Organization
mhGAP-HIG: Mental Health Gap Action Programme Intervention Guide
UNHCR: United Nations High Commissioner for Refugees
IOM: International Organization for Migration
MHPSS: Mental Health and Psychosocial Support
