# Supplementary material for: Common mental disorders in asylum seekers and refugees: umbrella review of prevalence and intervention studies
Source: Int J Ment Health Syst. 2017 Aug 25;11:51. doi: 10.1186/s13033-017-0156-0 (PMC5571637; doi:10.1186/s13033-017-0156-0)
Supplement: Supplementary file 1 — Additional file 1. Overall summary of the efficacy of interventions. [file 13033_2017_156_MOESM1_ESM.docx]

| Intervention | Outcome | Inactive | Active | Without control group |
| --- | --- | --- | --- | --- |
| NET | PTSD | ●●●●●●●● | ●●○ | 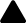 |
|  | Depression | ●●○○○ | ○○ | 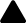 |
|  | Anxiety | ○ | ○ |  |
| KIDNET | PTSD | ● |  |  |
| EMDR | PTSD | ○ |  |  |
|  | Depression | ○ |  |  |
|  | Anxiety | ○ |  |  |
| CBT | PTSD | ◼◼●●●●○○ | ○ | 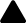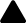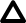 |
|  | Depression | ◻●●○○ | ○ | 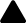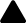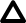 |
|  | Anxiety | ◼●●● | ○ | 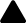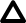 |
| Testimony therapy | PTSD |  |  | 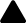 |
|  | Depression |  |  | 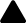 |
| Trauma-focused therapy | PTSD | ◼◼ | ○ | 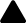 |
|  | Depression |  |  | 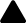 |
| Multimodal interventions | PTSD |  |  | 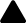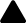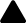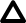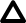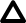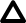 |
|  | Depression |  |  | 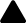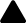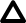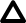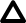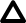 |
|  | Anxiety |  |  | 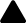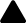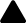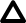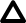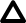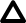 |
| Antidepressants | PTSD |  |  | 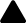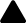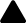 |
|  | Depression |  |  | 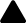 |
| Psychoactive medication+  Psychodinamic therapy | PTSD | ◼ |  |  |
| Abbreviations: NET= Narrative Exposure Therapy; KIDNET= Narrative Exposure Therapy for children; EMDR= Eye Movement Desensitisation and Reprocessing; CBT= Cognitive Behavioral Therapy; CPT= Cognitive Processing Therapy.  Legend:  ◼ Controlled Clinical Trial (CCT) showing a statistically significant positive effect  ◻ Controlled Clinical Trial (CCT) failing to show a statistically significant positive effect  ● Randomised Clinical Trial (RCT) showing a statistically significant positive effect  ○ Randomised Clinical Trial (RCT) failing to show a statistically significant positive effect  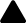 Study without control group showing a statistically significant positive effect  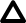 Study without control group failing to show a statistically significant positive effect | | | | |

**Additional file 1.** **Overall summary of the efficacy of interventions**
